# Inactivating Gene Expression with Antisense Modified Oligonucleotides

**DOI:** 10.32607/actanaturae.11522

**Published:** 2021

**Authors:** Sidney Altman, Carlos Angele-Martinez

**Affiliations:** Yale University New Haven CT USA, Arizona State University, Tempe AZUSA; Yale University New Haven CT USA

**Keywords:** modified nucleotides, gene expression, bacteria, Plasmodium falciparum, citrus plants

## Abstract

Modified nucleotides, including phosphoramidates and mesyl nucleotides, are very effective in inactivating gene expression in bacteria. *Gyr A *is the target gene in several organisms, including *Plasmodium falciparum*. Antisense reactions with bacteria infecting citrus plants are promising but incomplete. Human tissue culture cells assayed with a different target are also susceptible to the presence of mesyl oligonucleotides.

## INTRODUCTION

**Fig. 1:**
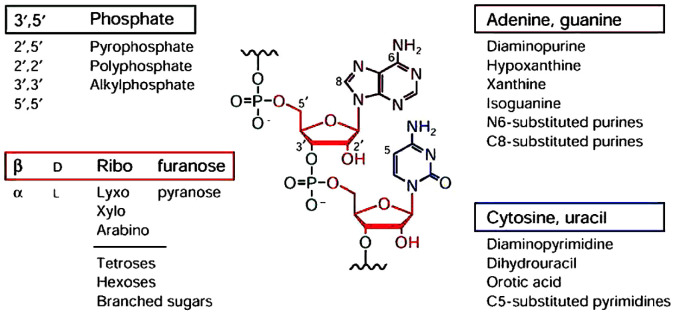
Schematic portrayal of the standard ribonucleotides with substitutes in different positions

**Fig. 2:**
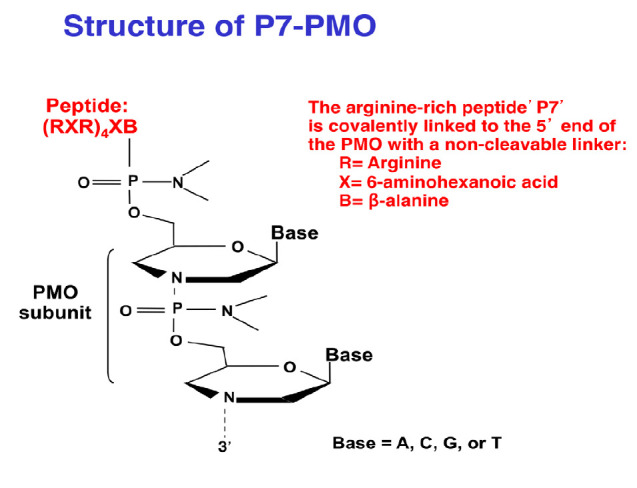
The portrayal of a phosphoramidate with a basic peptide attached to the 5’ end. Courtesy of Sarepta

Over several years, the research focused on RNase P has been relying on standard oligonucleotides A, C, U, G in RNA (*Fig. 1*). That was sufficient to probe both the function and structure of the enzyme in bacteria [1,, 2, 3, 4, 5]. However, once the focus switched to the study of the suppression of the activity of various genes, the advent of phosphoramidates (PMs; [6, 7]; see *Fig. 2*) and 2’OMe nucleotides, which have the advantage of being characterized by a higher membrane permeability and nuclease resistance compared to those of standard oligonucleotides, has led to a spate of attempts using modified oligonucleotides (MOs) as antisense oligonucleotides to turn gene expression off.

Various permutations of MOs (i.e., using different modified oligos at various positions in the antisense molecules) proved unsuccessful in gene inactivation studies. Fully modified MOs were nonspecifically lethal in living cells. When using modified oligonucleotides, the application of RNase P and a particular MO to target gene expression has the potential to kill the bacteria being studied (*gyr A*, the ultimate target; [8]) in lethal gene suppression studies. In the present report, critical metabolism functions were not extensively studied.

The target chosen was the essential gene *gyr A *[8], the aspecific target gene sequence in the gene being almost invariant in several bacteria (*Table 1*). An MO with a 5’ peptide attached (*Fig. 2*) was used to test most of the bacteria with the gyr A target. This oligonucleotide facilitated the import of MOs in bacteria.

**Table 1:**
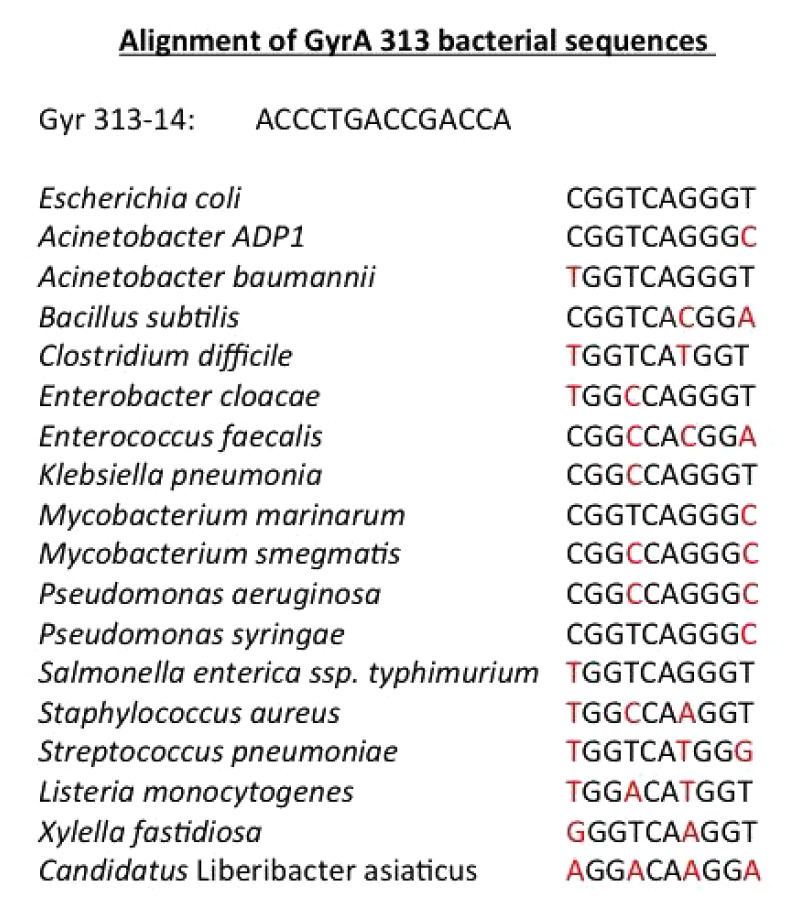
The portrayal of a phosphoramidate with a basic peptide attached to the 5’ end. Courtesy of Sarepta

**Fig. 3:**
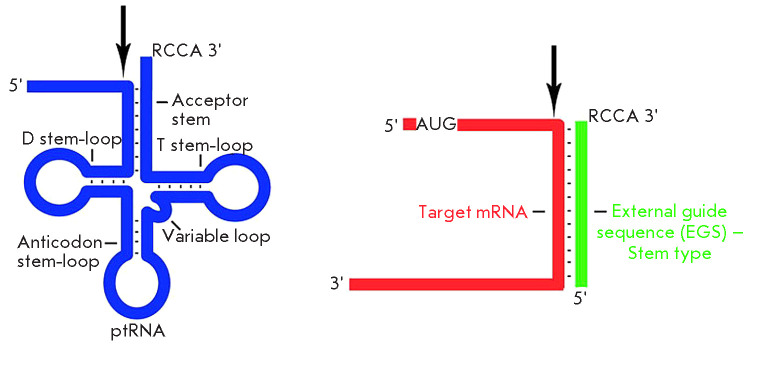
(Left) Schematic portrayal of a tRNA precursor with the arrow indicating the site of cleavage by RNase P (Right). Portrayal of a minimal substrate for RNase P

RNase P will cleave any oligonucleotide containing the 3’CCA sequence and at least one extra nucleotide at its 5’ end adjacent to the double-stranded region (For details please refer to *Fig. 3*) [9]. The initial experiments to suppress the activity of the genes that provide drug resistance (penicillin, chloramphenicol) used the known properties of RNase P (*Table 2*, [10, 11, 12]). The success of the new methodology is apparent: under the conditions used by us, *Streptococcus *and* Staphylococcus *were inhibited only to a level of 10– after 6 h of incubation at 37°C. Two major pathogens,* Yersinia pestis *and *Francisellatularensis*, assayed using MOs in a slightly different way, were also inhibited to a level of 40–50% [13, 14], but *gyr A *was not the target in the two cases, and these experiments were never pursued. The new methodology and the gyr A target can be employed to ensure a much lower survival rate under the conditions used.

**Fig. 4:**
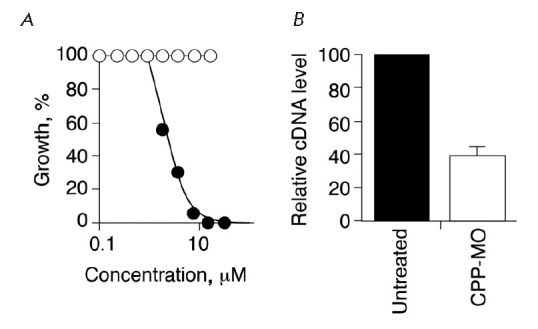
Survival of *P. falciparum *in red blood cells after treatment with an antisense oligonucleotide

**Fig. 5:**
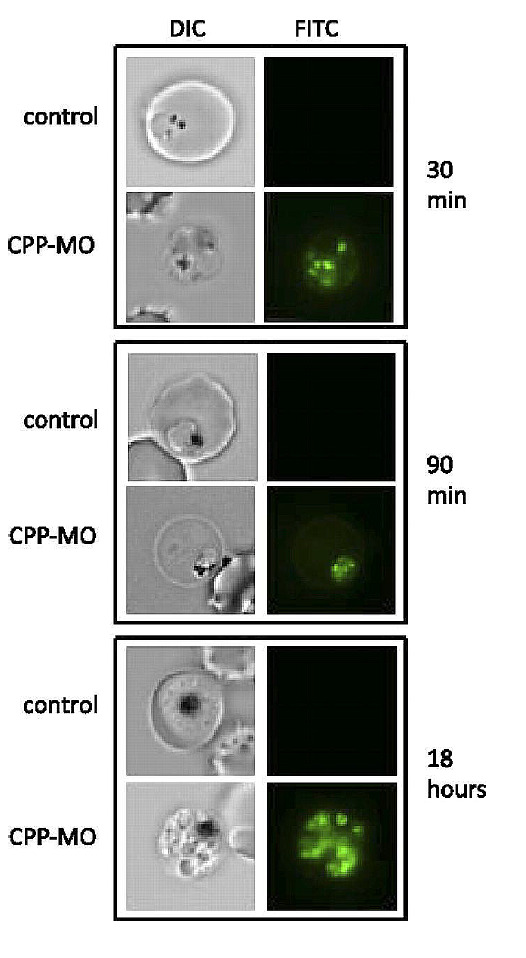
Development of *P. falciparum *in red blood cells after treatment with an antisense oligonucleotide. Fluorescence microscopy images are shown on the right-hand side of the figure. No lysis of red blood cells by the fluorescent dye occurred

**Fig. 6:**
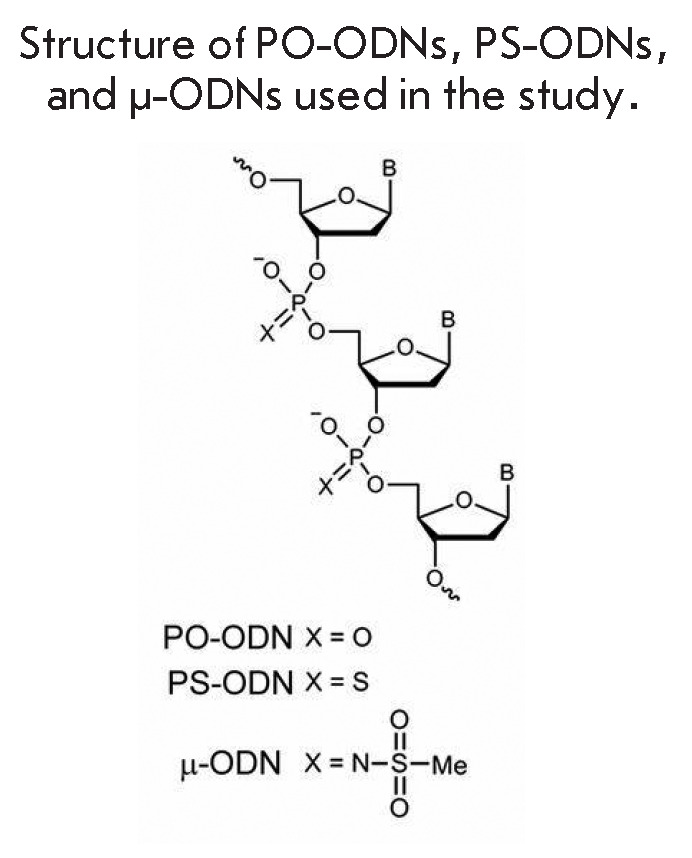
Schematic portrayal of an oligonucleotide with substitutions indicated, including the mesyl group

*Table 1*lists several sequences in bacteria that are complements to the *E. coli gyr A *sequence as a target. It is noteworthy that there are several W-C mismatches in some bacteria, but at the most three proved successful in the experiments that were performed. The viability of bacteria infected with an appropriate MO decreased from 3 to 6 orders of magnitude after the incubation with the MO at 37°C for 6 h (*Table 2*; *Fig. 2* and *Fig. 4*). Several bacteria responsible for acute human infections (*Streptococcus *and *Staphylococcus*) can be inactivated in the way noted above. A MO with an attached peptide facilitated the penetration of MO into bacteria, as shown in *Table 2* [15, 16] and *Fig. 4*. Subsequently, the *in vitro *inactivation of *P. falciparum *in red blood cells was tested again, with targeting of the *gyr A *gene of this organism [17, 18]. These experiments have been successful (*Fig. 5* and *Fig. 6*). Note that the development of the *P. falciparum* is clearly inhibited (50%) at a MO concentration of 0.5 ѓKg/ml (*Fig. 6*). The MO used was effective against* P. falciparum *cells, being resistant to different drugs (arteminisin, etc.), as well as against the normal parasite. The prospect of using the MO as an anti-malarial therapy remains to be explored.

**Table 2:**
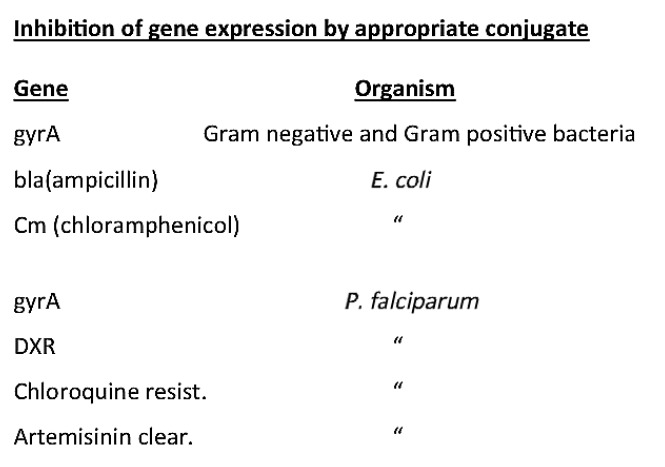
Inactivation of various genes using the antisense technology (Bacteria and P. falciparum were exposed to an antisense molecule (~ 5 ug/ml) at 37°C for 6 hrs. Please see the text for the details.)

The general method failed to work in one experiment in mice where the amount of MO was inadequate and no lipofectamine was used to aid cell penetration of the MO and additionally there was no taking into consideration the cost of MO synthesis and the enzymatic subunit of *Escherichia coli*, RNase P. Research then subsequently shifted to bacterial infection in plants (e.g., inactivation of citrus plants by infecting bacteria).

The citrus industry in the U.S. has suffered devastating losses from infection by the insect *Diaphorinacitri* carrying a bacteria that renders saplings and trees unable to produce fruit [19]. *Table 1* shows the sequence of the complement of the *Wolbachia gyrA *gene. Several* Wolbachia *species can infect citrus saplings. RNase P can be isolated from *D. melanogaster *S2 cells *in vitro* to indicate the ability of similar flies to produce the enzyme. It would cleave tRNA precursors and M1 RNA in separate experiments. To show cleavage of the *gyr A *sequence, the *Wolbachia *sequence (*Table 1*), the MO, and the *gyr *RNA were exposed to M1 RNA, as well as to the purified *E. coli *RNase P. Preliminary analysis of the M1 RNA MO reactions with *gyrA *RNA, with low levels of radioactivity, indicated that some successful cleavage took place. These experiments must be repeated, along with the reactions with the *Wolbachia gyr A *sequence, which has only three mismatches with* E. coli gyr A*. If these reactions succeed, confirming the lethality of *Wolbachia in vitro*, a method of administration to hundreds or thousands of saplings must still be developed.

The *C. liberibacterasiaticus*, another factor in infecting saplings, has four mismatches in its *gyr A *gene compared to the one in *E. coli* (*Table 1*), an indication that complementarity would not be a valid counterpart in our experiments using MOs (*Table 1*).

**Fig. 7:**
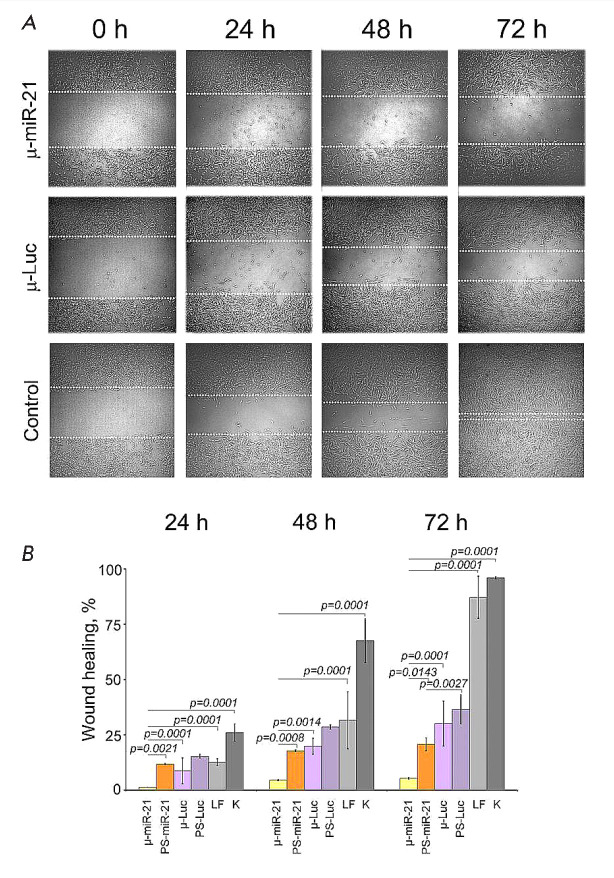
The kinetics of wound healing with different mesyloligonculeotides. Zero time points were identical or corresponded to whole colonies. *A*. The image of the colonies at different time points. *B*. The kinetics of wound healing. K is the control. Other non-specific targets are also indicated

Stetsenko et al. have recently synthesized a new modified oligonucleotide named mesyl MO (*Fig. 6* [20]) that is DNA-based and is more effective in inactivating gene expression. Its lethality characteristics are increased in comparison with previously designed modified oligos. RNase H attacks the DNA-miR21 hybrid. This new MO is much less successful in aiding cells separated by a scratch test to migrate during wound healing than cells transfected with other MO oligos (*Fig. 7*). This method of synthesizing mMO should be tested for inactivating the expression of the various genes mentioned in this paper. The new mesyl oligo is much more resistant to nonspecific nuclease degradation than other oligos and is 22 nts long, making it unique in human cells. A lipofectamine-driven encapsulation provides entry of this oligonucleotide into the cells in tissue culture. The targets listed in *Fig. 7* include miR21, specific to the new MO, and other non-specific targets. At zero time, all the samples showed intact colonies.

During the past few years, antisense oligonucleotides have been used to inhibit the function of various molecules in tissue culture cells. The prospect for designing new antibiotics has not been quite as bright as was promised by the industry, but some results have been achieved with derivatives of aminoglycosides and a few other molecules. Derivatives of hammerhead ribozymes are still being studied as potential mechanisms of gene inactivation. The design of small pieces of RNA with distinct three-dimensional structures as gene inactivation inhibitors is in progress. Antisense has also been used to alter splicing reactions. In one case, antisense molecules have been productively used as therapy in cases of Duchenne muscular dystrophy [6]. The future of antisense molecules, modified or otherwise, appears bright.
